# The Characteristics and Expression Profile of Transferrin in the Accessory Nidamental Gland of the Bigfin Reef Squid during Bacteria Transmission

**DOI:** 10.1038/s41598-019-56584-8

**Published:** 2019-12-27

**Authors:** Hau-Wen Li, Chih Chen, Wei-Lun Kuo, Chien-Ju Lin, Ching-Fong Chang, Guan-Chung Wu

**Affiliations:** 1 Department of Aquaculture, National Taiwan Ocean University, Keelung, Taiwan; 2 Center of Excellence for the Oceans, National Taiwan Ocean University, Keelung, Taiwan

**Keywords:** Innate immunity, Cellular microbiology

## Abstract

The accessory nidamental gland (ANG) is a female reproductive organ found in most squid and cuttlefish that contains a consortium of bacteria. These symbiotic bacteria are transmitted from the marine environment and selected by the host through an unknown mechanism. In animals, a common antimicrobial mechanism of innate immunity is iron sequestration, which is based on the development of transferrin (TF)-like proteins. To understand this mechanism of host-microbe interaction, we attempted to characterize the role of transferrin in bigfin reef squid (*Sepioteuthis lessoniana*) during bacterial transmission. qPCR analysis showed that *Tf* was exclusively expressed in the outer layer of ANG,and this was confirmed by *in situ* hybridization, which showed that *Tf* was localized in the outer epithelial cell layer of the ANG. Western blot analysis indicated that TF is a soluble glycoprotein. Immunohistochemical staining also showed that TF is localized in the outer epithelial cell layer of the ANG and that it is mainly expressed in the outer layer during ANG growth. These results suggest that robust *Tf* mRNA and TF protein expression in the outer layer of the ANG plays an important role in microbe selection by the host during bacterial transmission.

## Introduction

The eggs of most cephalopods are enclosed within a capsule composed of different egg membranes produce by oviducal glands and nidamental glands^1^. In addition, a pair of accessory nidamental glands (ANGs) is found in most squid and cuttlefish (Decapodiformes) but is absent in some squid (Oegopsidae), and all octopuses (Octopodiformes) and nautiluses (Nautiloidea)^2^. The color of the ANG changes from white to orange and red during sexual maturation in female squid and cuttlefish. These colors represent accumulation carotenoids synthesized by bacteria in the ANG^3^. Antimicrobial activity is found in extracts of the ANG in squid^4,5^ and cuttlefish^6^, which is interesting in light of the highly similar bacterial composition of the egg jelly coat and ANG in these cephalopods^7,8^. The ANG may play an important role in bacterial delivery from the parent to the egg capsule by preventing contamination from microorganisms in the benthic environment^9^. In squid and cuttlefish, at least some symbiotic bacteria are delivered from the marine environment and selected by the host^10^. For example, the mechanism of bacterial selection and colonization is well established in the light organ of the Hawaiian bobtail squid, *Euprymna scolopes*^11,12^. However, a comprehensive understanding of bacterial selection during bacterial transmission to and colonization of the ANG is lacking.

Iron is an essential element for the growth and development of all living organisms. In animals, a common antimicrobial mechanisms of innate immunity are based on the development of transferrin-like proteins with high affinity for trivalent iron (Fe^3+^)^13,14^. Three *transferrin*-like genes are found in vertebrates, including *transferrin* (*TF*), *lactotransferrin* (*LTF*), and *melanotransferrin* (*MELTF*)^15^. Based on amino acid sequence, location, and putative function, the TF family has been divided into two branches: one represented by membrane MELTF and soluble TF (serotransferrin and ovotransferrin) and the other by LTF^15^. Serotransferrin and ovotransferrin are encoded by the *TF* gene and are expressed in both the liver and oviduct in birds^16^ and reptiles^17^. While the primary function of serotransferrin is iron transport, ovotransferrin plays an anti-microbial role in egg albumin in birds^13,18^ and reptiles^19^. LTF has similar functions to ovotransferrin and is found in most milk and tear secretions in mammals, where its role is to inhibit the proliferation of invading microorganisms^20^. The precise function of MELTF remains unknown. Bacterial challenge causes a significant increase in *TF*/*Tf*/*tf* expression in fish^21–23^, amphioxus^24^, crustaceans^25,26^, insects^27^, and mollusks^28^. Thus, TF may play an important antimicrobial role through iron sequestration in teleost and invertebrates.

To elucidate the potential role of TF in the ANG of cephalopods during bacterial transmission, we cloned a *Tf* gene from the bigfin reef squid (also called oval squid, *Sepioteuthis lessoniana*). To visulize the localization of TF in the ANG of bigfin reef squid, we made a TF-specific antibody and demonstrated the presence of *Tf*-expressing cells and TF itself in the outer epithelial cell layer of the ANG. We also found that TF is mainly distributed in the outer epithelial cell layer of the ANG during the developmental stage in which bacterial colonization occurs. TF may, therefore, play an important role in the primary phase of bacterial selection in the ANG of bigfin reef squid.

## Results

### Molecular identification and phylogenetics of bigfin reef squid *Tf* gene

**Figure 1:**
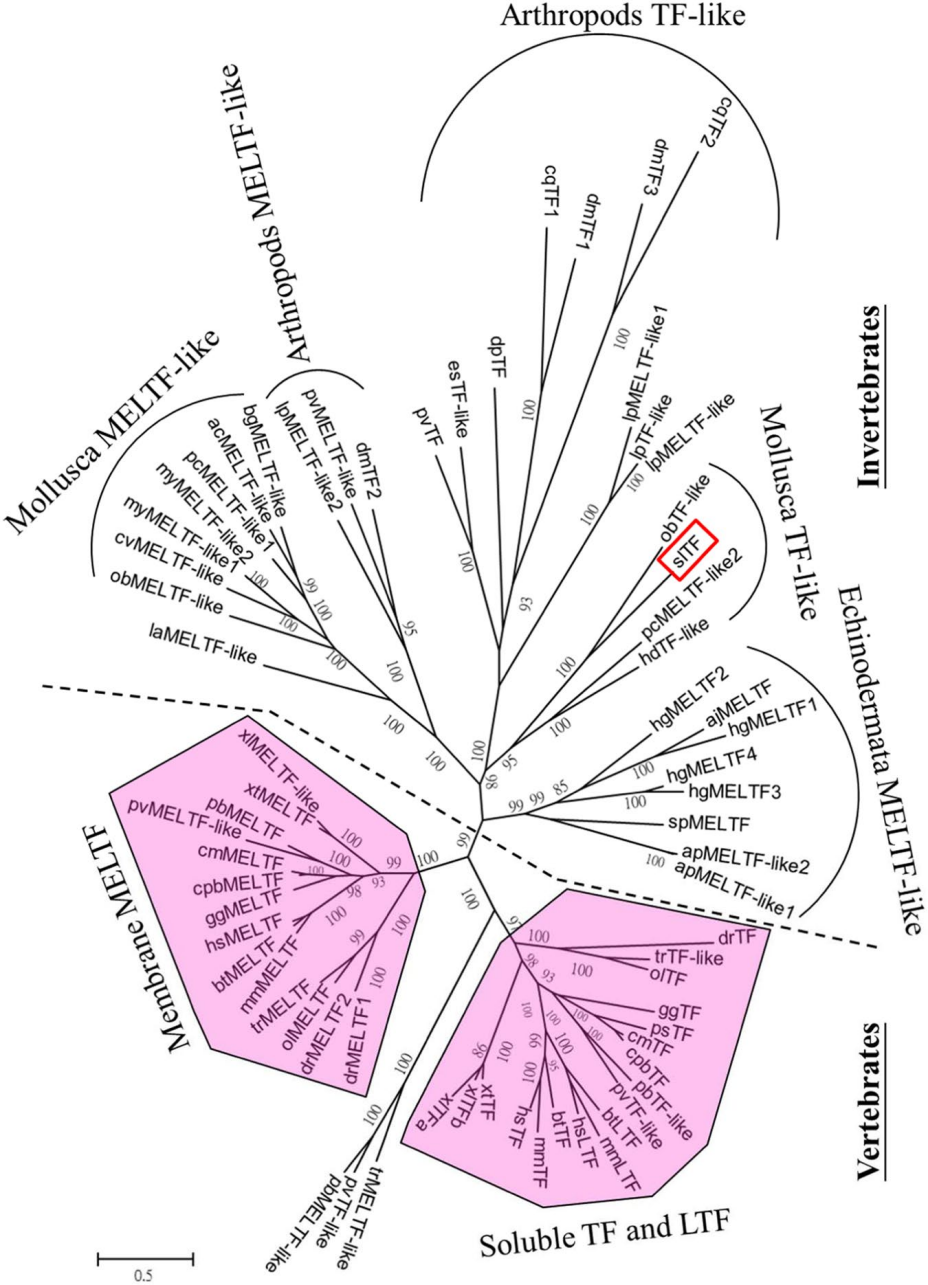
Phylogenetic tree comparing the amino acid sequences of the open reading grame of the TF family from various taxa. The tree was constructed by using PhyML with the LG model of substitution, combined with the neighbor-joining interchange method on the Seaview browser. The number at each node represents the bootstrap probability (% from 1000 replicates). Branches correspond to bootstrap values of 50% and higher. The TF of bigfin reef squid (slTF) is enclosed in a red box. Two branches, one for membrane MELTF and soluble TF, and the other for LTF branch, are highlighted in pink polygons. The names and accession numbers of the sequences obtained in the analysis are listed in Supplemental Table 1.

According to the deduced amino acid sequences in the transcriptome database of the ANG of immature females, a *Tf*-like gene was found in the bigfin reef squid through a local blast on CLC Genome Workbench 8.0, and the sequence of the *Tf*-like gene was determined by cDNA cloning. The *Tf*-like gene had an open reading frame encoding 688 amino acid residues. Its deduced amino acid showed conserved features of TF, including the N-terminal (26–329 aa) and C-terminal (330–669) TF domains, connected by a very short peptide span. A phylogenetic analysis of this *Tf*-like gene and deduced sequences of TF family members from different species showed that the *Tf*-like gene (named *Tf*) was clustered with *Tf*-like and *Meltf*-like genes in mollusks (Fig. 1).

### Expression profiles of *Tf* during ANG growth

**Figure 2:**
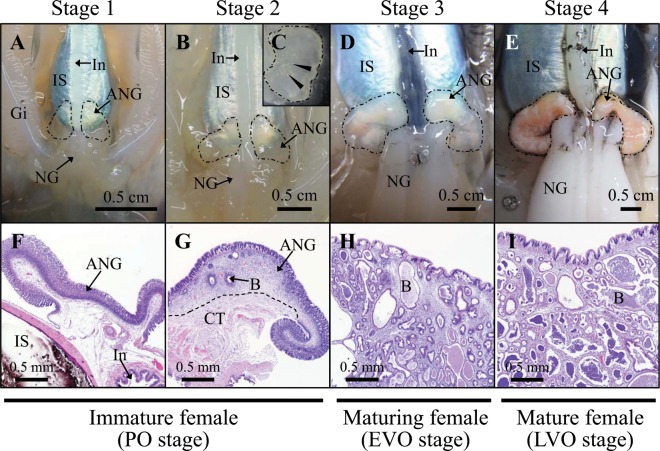
Morphological and histological changes during ANG growth in female bigfin reef squid. Morphological changes: (**A**) juvenile squid with a colorless ANG (stage 1); (**B**,**C**) immature squid with a colorless ANG with several white spots (stage 2); (**D**) maturing squid with a white/light-orange ANG (stage 3); (**E**) mature squid with a pigmented ANG (stage 4). Histological changes: (**F**) juvenile squid with no visible bacterial colonies in ANG (stage 1); (**G**) immature squid with several bacterial colonies in the ANG (stage 2); (**H**,**I**) bacterial colonies numerous and spread in the ANG (stages 3 4, respectively). ANG, accessory nidamental gland; B, bacterial colony; CT, connective tissue; Gi, gill; In, intestine; IS, ink sac, NG, nidamental gland.

**Figure 3:**
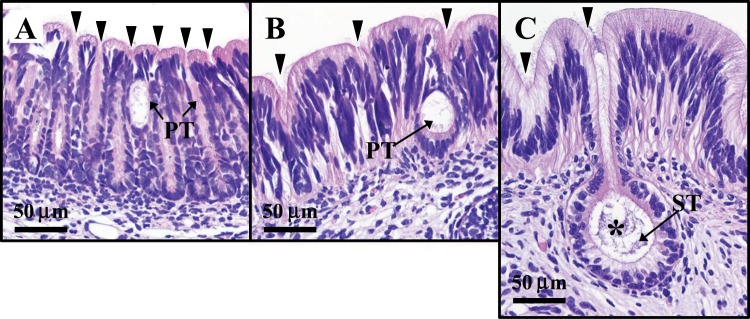
Histological evidence of bacterial transmission and colonization of the outer epithelial cell layer of the ANG. (**A**) Vesicle-like structures in the primary tubule; (**B**) vesicle-like structures anchored to the basement of the epithelial cell layer; (**C**) bacteria in invaginated precursors of secondary tubules. The asterisk (*) indicates a bacterial aggregation. The black arrowheads indicates the necks of the tubules. PT, primordial tubule; ST, secondary tubule.

**Figure 4:**
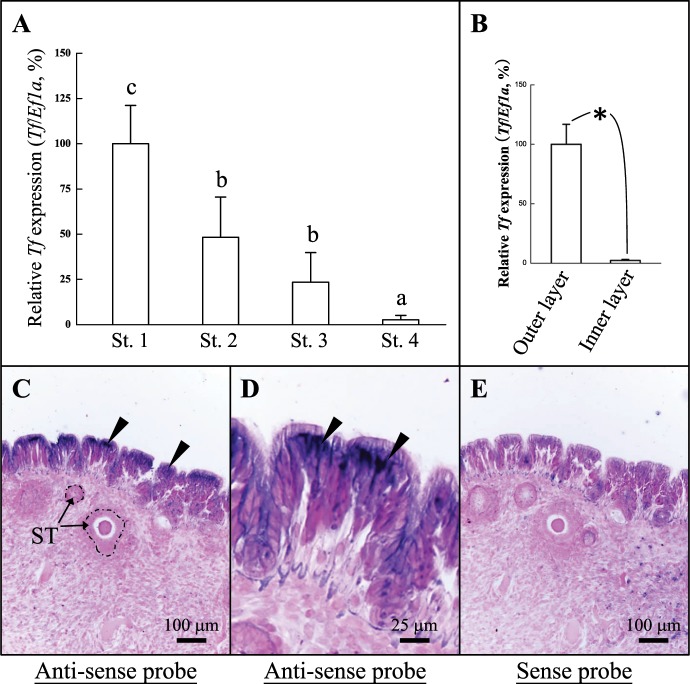
*Tf* gene expression profile and location of expression during ANG growth. Gene expression patterns were ascertained at four developmental stages of the ANG distinguished by histological criteria: juvenile stage 1 (n = 5), immature stage 2 (n = 11), maturing stage 3 (n = 7), and mature stage 4 (n = 8). With developmental stages. (**A**) Expression of *Tf* during ANG growth as analyzed by qPCR. (**B**) The expression of *Tf* in the outer and inner layer of ANG as analyzed by qPCR. (**C**,**D**) *Tf* mRNA expression in outer epithelial cell layer of ANG was detected by *in situ* hybridization (ISH) in immature female squid. (**E**) The reference of *Tf* expression was detected by the sense probe of *Tf*. In qPCR, differences between stages or tissues were normalized with respect to *Ef1a* gene expression, and the highest relative value of Tf was defined as 100%. Lower-case letters indicate significant differences by one-way ANOVA and Games-Howell test (*P* < 0.05). The asterisk indicates a significant difference according to Student’s t-test (*P* < 0.05). ST, secondary tubule. The black arrowheads denote sites of *Tf* expression.

Gene transcript levels were assayed of different developmental stages of the ANG of female squid. Recognized on the basis of morphological and histological characteristics (Table 1): juvenile (primary oocyte stage) squid with a colorless ANG without bacterial colony (stage 1; Fig. 2A,F), immature squid (previtellogenic oocyte stage) with a colorless ANG with bacterial colonies (stage 2; Fig. 2B,C,G), maturing (early vitellogenic oocyte stage) squid with a white/light-orange ANG with large numbers of bacterial colonies (stage 3; Fig. 2D,H), and mature (late vitellogenic oocyte stage) squid with a pigmented ANG with large numbers of bacterial colonies (stage 4; Fig. 2E,I). Histological observations showed how bacterial transmission and colonization took place. First, the outer epithelial cell layers of the ANG became invaginated, forming the primordial tubules, which were lobular structures open to the mantle cavity (Fig. 3A,B). Second, columnar epithelia were observed in the secondary lobules, which were filled with bacteria (Fig. 3C). To summarize, colonizing bacteria migrated from the mantle cavity to the ANG along the epithelial cell layers during ANG growth. According to the qPCR results of *Tf* expression in various tissues of mature female squid, *Tf* was predominantly expressed in the tentacles and hemocytes but was also detected in the mantle, optic lobes, brain, stomach, hepatopancreas, gills, heart, ovary, oviduct, oviducal gland, nidamental gland, and ANG (Fig. S1). Furthermore, qPCR analysis showed that *Tf* expression levels were high in stage 1 of ANG development and that expression levels decreased significantly in stages 2–4 (Fig. 4A).

**Table 1:** Characteristics of sampled squids.

	Sample No.	ML (cm)	BW (g)	GW (g)	GSI	Gonadal stage	Reproductive phase
Stage 1	5	14.5 ± 1.2	184.7 ± 34.2	<0.1	nd	PO	Immature female
Stage 2	11	19.3 ± 3.3	403.5 ± 198.5	0.4 ± 0.2	0.12 ± 0.07	PO	Immature female
Stage 3	7	24.4 ± 1.8	787.7 ± 185.1	0.9 ± 0.3	0.11 ± 0.04	PVO	Maturing female
Stage 4	8	27.7 ± 3.0	1127.5 ± 362.2	11.9 ± 7.1	0.99 ± 0.44	VO	Mature female

No, number; ML, mantle length; BW, body weight; GW, gonad weight; GSI, gonadosomatic index (%); PO, primary oocyte; PVO, previtellogenic oocyte; VO, vitellogenic oocyte.

### Localization of *Tf* expression

To analyze the distribution of *Tf* expression in ANG, outer and inner layers of ANGs of mature female squid (stage 4 of ANG) were isolated by stereomicroscope and analyzed separately. Histological examination confirmed that the outer layer had been completely removed (Fig. S2). qPCR results showed that the outer epithelial cell layer had higher *Tf* expression than the inner layer of the ANG in mature females (stage 4; Fig. 4B). ISH with antisense probes of *Tf* were used to analyze the gene transcripts in female squid. This showed *Tf* mRNA expression in the outer epithelial cell layer of the ANG, but no signal was observed in the organ’s connective tissue or in the columnar epithelia of the secondary tubules (Fig. 4C,D). Furthermore, no signal was observed from sense probes for *Tf* (Fig. 4E).

### Specificity of an anti-TF antibody

**Figure 5:**
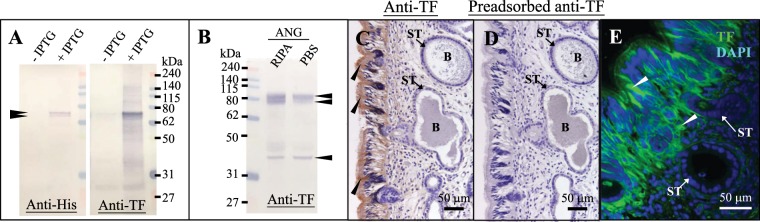
Antibody specificity of TF protein in ANG. rTF and protein extracts of ANGs were used to analyze the antibody specificity. (**A**) rTF not detected by anti-histidine tag antibody (Anti-His) and anti-TF antibody (Anti-TF) in reference (−IPTG) but detected in IPTG-induced samples (+IPTG). (**B**) WB detected the signals of TF by anti-TF antibody in ANGs. (**C**,**D**) IHC staining of TF in the ANG using anti-TF antibody and antigen-adsorbed anti-TF antibody. (**E**) IF staining of TF in the ANG using anti-TF antibody. The black arrowhead denotes the TF signals by WB and TF expression by IHC staining. The white arrowhead denotes TF expression by IF staining. B, bacteria; ST, secondary tubule.

Based on the ExPASy website (http://web.expasy.org/compute_pi/), after cleavage of the signal peptide, TF of bigfin reef squid had a theoretical size of 76 kDa. Furthermore, the N-terminal (26–329 aa) and C-terminal (330–669) fragments had theoretical size of 30.6 kDa and 40 kDa, respectively. The specificity of the TF antibody generated in this study was confirmed by Western blot analysis using recombinant TF (rTF) and ANG extracts. Immunoblots of IPTG-induced bacterial protein extracts revealed two rTFs, 72-kDa and 75-kDa using the anti-histidine tag antibody and anti-TF antibody (Figs. 5A and S3). No immunoblot signal in non-induced (without IPTG) bacterial protein extracts was observed using the same antibodies (Fig. 5A). Immunoblot analysis of ANG extracts revealed three TF proteins of 80-, 78-, and 38-kDa using the anti-TF antibody (Figs. 5B and S3). The immunoblotting signals of TF were significantly reduced when antigen-preadsorbed serum of the anti-TF antibody was used (Figure S). Thus, the anti-TF antibody only recognized the TF protein in ANG. These detected squid TF proteins (80-kDa and 78-kDa) were larger than the respective theoretical sizes, but glycosylation may have affected the molecular weight. Moreover, a TF fragment (38-kDa) shows that the anti-TF antibody (the antigenic epitope of TF is located on the C-terminal fragment) could not recognize the N-terminal fragment of TF.

### TF distribution in the outer epithelial cell layer of the ANG during ANG growth

**Figure 6:**
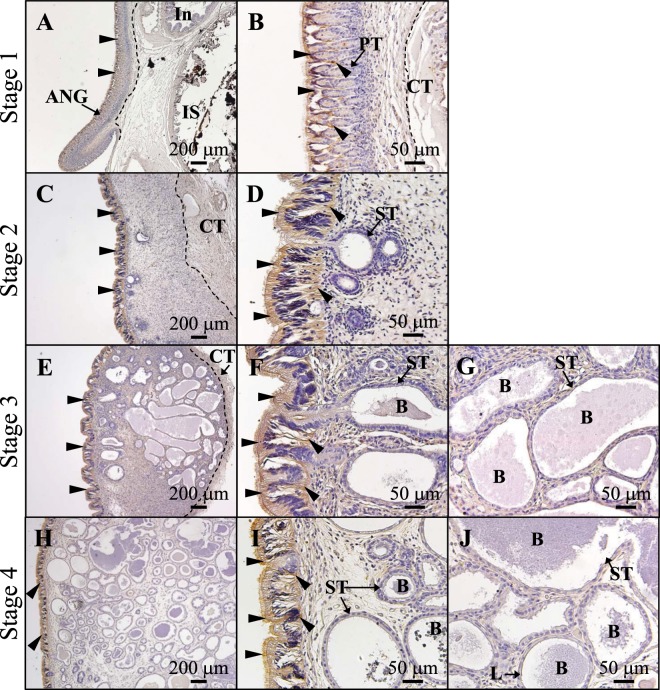
TF protein localization patterns during ANG growth. Histology of ANGs, four different ANG stages were used to analyze the protein expression pattern by IHC staining, including stage 1 in juvenile squid (**A**,**B**) stage 2 in immature squid (**C**,**D**) stage 3 in maturing squid (**E**–**G**) and stage 4 in mature squid. (**H**–**J**) Black arrowhead indicate site of TF expression. ANG, accessory nidamental gland; B, bacteria; CT, connective tissue; In, intestine; IS, Ink sac; PT, primary tubule; ST, secondary tubule.

IHC and IF were used to analyze the protein expression in female squid. Strong TF signals were found in the outer epithelial cell layer of the ANG but not the columnar epithelium of the secondary lobules (Fig. 5C). No signal was observed from the antigen-preadsorbed serum of the anti-TF antibody (Fig. 5D). Furthermore, IF staining showed that TF signals were confined to the cytoplasm of epithelial cells (Fig. 5E). To summarize, *TF* mRNA and TF protein were exclusively expressed in the outer epithelial cell layer of the ANG.IHC staining showed TF expression in the outer epithelial cell layer of the ANG at stage 1 (Fig. 6A,B), and mainly in the outer layer at stage 2 (Fig. 6C,D), stage 3 (Fig. 6E,F), and stage 4 (Fig. 6H,I). No TF expression was found in the secondary tubules at stage 2 (Fig. 6C,D), stage 3 (Fig. 6E,F,G), or stage 4 (Fig. 6H,I,J). The specificity of anti-TF antibody was confirmed using the antigen-preadsorbed anti-TF antibody in different stages of ANG. Signal from this were absent or slight, in contrast to the strong signals observed using the anti-TF antibody (Fig. S5).

## Discussion

Because bacteria were observed on the surface (outer epithelial layer) of the ANG at stage 1 (Figs. 2 and 3), but later appeared within the invaginating primordial and secondary tubules at stage 2 (Figs. 2 and 3), our data support the hypothesis that the ANG houses a bacterial consortium that is horizontally transmitted from the environmental bacterial community. The most abundant bacterial taxa of cephalopod ANGs are gram-negative *Alphaproteobacteria*^7,8,29–31^, but the make-up of the bacterial community varies among cephalopod species. In loliginid squid and cuttlefish, a combination of *Alphaproteobacteria* and *Gammaproteobacteria* is dominant^8,29,31^, whereas the dominant bacterial taxa in bobtail squid are a combination of *Alphaproteobacteria* and *Verrucomicrobia*^7,30^. Similar to other loliginid squids, the ANG of bigfin reef squid had a large *Alphaproteobacteria* contingent, comprising a *Rhodobacter* clade, a *Roseobacter* clade, and an *Agrobacterium-Silicibacter* clade^8^. Furthermore, fluorescence *in situ* hybridization (FISH) analyses showed that the tubules of the ANG are dominated by single taxonomic groups^29,30^. During light organ colonization of the Hawaiian bobtail squid by bacteria (*Vibrio fischeri*), a mucus secretion derived from host epithelial cell provides a nonspecific surface upon which environmental bacteria form a aggregate and biofilm in the initial step^11^. In the second step, colonization by symbiotic *V*. *fischeri* inhibits this mucus secretion^11^. Then the *V*. *fischeri*-secreted outer membrane vesicles trigger an irreversible program of light organ development in the host^32^. Microbe-associated molecular patterns (lipopolysaccharide and tracheal cytotoxin) can also induced light organ development in the Hawaiian bobtail squid^33,34^, and the presence *Vibrio fischeri* in the light organ can enhance the gene expression of the cellular innate immune system in this squid’s hemocytes^35^. The cellular innate immune system also plays a role in the cephalopod ANG, as exemplified by the immune-related pfam domains in Hawaiian bobtail squid^36^ and thr Toll/NF-kB family in cuttlefish (*Sepia officinalis*)^37^. Taken together, these results suggest that the establishment and maintenance of the bacterial consortium in the ANG is an intricate process governed by host selection, consisting of initial bacterial transmission under universal selection and later bacterial colonization under independent selection. Based on amino acid sequence, location, and putative function, the TF family has been divided into two branches: a branch including membrane MELTF and soluble TF branch (serotransferrin and ovotransferrin), and an LTF branch^15^. *Tf* was cloned in bigfin reef squid in this study. According to our phylogenetic analysis, this TF was clustered with TF-like and MELTF-like in mollusks (Fig. 1). WB analysis showed that the molecular weight of TF was higher in ANG-extracts than in rTF (Fig. 5), a difference possibly caused by the glycosylation of TF. Moreover, WB analysis of TF showed the same proteolytic patterns of TF in the RIPA- and PBS-extracts, which shows that TF is localized in the cytoplasm. Taken together, our results demonstrate that the TF of bigfin reef squid is a soluble glycoprotein member of the transferrin family.

The low levels of *Tf* expression during ANG growth found by qPCR analysis may be due to the reduced proportion of the outer layer of the ANG in the whole organ as its development processes. The primary function of serotransferrin is iron transport, but ovotransferrin play an anti-microbial role in egg albumin in birds^13,18^ and reptiles^17^. LTF has similar functions to ovotransferrin and is found in most milk and tear secretions in mammals^20^. Bacterial challenge causes a significant increase in *tf*/*Tf* expression in fish^21–23^, amphioxus^24^, crustaceans^25,26^, and mollusks^28^. Levels of iron in the serum and hemolymph are decreased significantly with heightened TF expression after bacterial challenge in Wuchang bream^22^ and tobacco hawk moth^27^. Transferrin-like genes/proteins also found in the light organ (immune organ) of Hawaiian bobtail squid^38^. In mammals, bacterial iron availability is limited by mucosal secretion of host iron proteins to prevent dissemination of pathogens and to enhance the symbiosis of resident bacteria in the intestine^39^. Thus, TF may play an important role in the innate immune response in metazoans through the reduction of iron levels. In summary, the high level of *Tf* expression in hemocytes in bigfin reef squid may have the conserved function of reducing the iron level in the plasma through the evolution. In addition, high expression of *Tf* was found between the tentacles of bigfin reef squid in the present study and in the mantle of disk abalone^28^. These results suggest that TF may have a conserved antimicrobial role.

TF and LTF are also important sources of iron acquisition for Gram-negative bacterial pathogens in the mammalian intestine^40^. Bacteria have evolved numerous mechanisms to acquire iron from the host environment and iron-binding proteins. These mechanisms include siderophore-mediated transport, direct import through divalent metal transporters, and direct piracy from iron-bound host proteins^39^. Siderophores are small iron-chelating molecules produced by microbes for iron scavenging^41^. The capacity to synthesize them varies among different bacteria. Most members of the *Reseobacter* clad do not contain genes for sidetophore synthesis, but several species of this clade found in the ANG did contain siderophore systhesis genes and are thus adapted to iron scavenging^42^. In the mammalian intestine, siderophore production by bacteria depends on environmental factors including pH, oxygenation, and carbon source^43^. LF receptors of bacteria have another function, protection against the host’s cationic antimicrobial peptides^40^. Thus, the ability of siderophore-mediated bacteria to acquire iron within the host contributes to bacterial niche selection and can also shape bacterial community dynamics and host-microbial interaction^39^. Taken together, our data suggests that high *Tf* mRNA and TF protein expression in the outer layer of the ANG may play an important role in the primary phase of bacterial selection in this organ in bigfin reef squid.

We demonstrated that *Tf* showed higher levels of expression in the squid’s hemocytes, tentacles, and outer layer of the ANG than in other tissues and data indicate that TF is a soluble glycoprotein localized in the outer epithelial cell layer of the ANG, where it is formed *de novo* through expression of *Tf* mRNA. This data suggests that *Tf* plays an important role in universal selection of bacteria by the host during bacterial transmission. Further studies of *Tf* regulation in bigfin reef squid are required for a better understanding of the regulatory mechanisms of host-bacteria interaction in cephalopods.

## Methods

### Squid collection

Bigfin reef squid were purchased from a fisherman on Heping Island, Keelung City, Taiwan. These Squids were collected by jigging off the northeast coast of Taiwan. The squid were transferred to a seawater tank (2.5 tons) and left there for 2 hours. The squid were anesthetized in seawater containing 5% ethanol at room temperature. After tissue collection, the squid were euthanized by cutting off the head. All procedures and investigations were approved by the National Taiwan Ocean University Institutional Animal Care and Use Committee and were performed in accordance with standard guide lines.

### Separation of outer layer and inner layer of ANG

The outer and inner layer of ANGs from mature female squid (stage 4, n = 4) were isolated by tweezers under a stereomicroscope and used for RNA analysis.

### Tissue histology

Hematoxylin and eosin (H&E) staining was performed as described previously^44^. The ANGs were fixed with 4% paraformaldehyde in PBS at 4 °C for 16 hours, then dehydrated in methanol and stored at −20 °C. Rehydrated ANGs were transferred from methanol to ethanol and then embedded in paraffin. Sections (6 μm in thickness) were rehydrated with PBS, treated with HistoVT One (Nacalai Tesque), and then stained with hematoxylin and eosin. The ANGs of female squid were examined histologically. The ovarian stagewas determined by the size of the oocytes, in accord with our previous study^44^.

### Total RNA extraction and cDNA synthesis

After ANG status of all squid had been determined by histology, as listed in Table 1. Thirty-one female squid at different developmental stages were used to analyze the mRNA expression profile during ANG growth. Four different stages of the ANG were used to ascertain gene expression patterns, including a juvenile stage (stage 1, n = 5), an immature stage (stage 2, n = 11), a maturing stage (stage 3, n = 7), and a mature stage (stage 4, n = 8). ANGs were homogenized in TRIzol reagent (Invitrogen). Extraction of total RNA was performed as the manufacturer’s protocol. The quantity and quality of total RNA were determined by NanoDrop^TM^ 1000 spectrophotometer (Thermo Fisher Scientific) and gel electrophoresis, respectively. First-strand cDNA was synthesized from 1 μg total RNA with oligo(dT)_12–18_ primers (Promega) by Superscript III reverse transcriptase (Invitrogen).

### Cloning of the bigfin reef squid *Tf* gene

The ANG from an immature (stage 2) female squid was used to isolate total RNA as described above. The cDNA synthesis, cDNA library construction, and illumine sequencing were done by Welgene, Inc. (New Taipei, Taiwan). Paired-end sequencing (150 bp) was performed on a HiSeq 2000 sequencer (Illumina). A *de novo* transcriptome assembly was performed using CLC Genome Workbench 8.0 according to the manufacturer’s protocol. This draft transcriptomic database of the ANG (in preparation) was used to obtain a fragment of the target gene. A local blast on CLC Genome Workbench 8.0 was used to obtain the homolog of *Tf* in bigfin reef squid. The sequence of *Tf* was confirmed by cloning, using PCR primers (5′-CTTACCAGGCTCAACTATCATACA-3′ and 5′-GTCATCATCATCATCATCAACAAAGA-3′) designed based on the nucleotide sequence of the draft transcriptomic database of ANGs. A full-length (containing ORF) cDNA sequence of *Tf* (GenBank accession No. MK875790) was obtained in this study.

### Sequence alignment and phylogenetic analysis

For phylogenetic analysis, a subset of transferrin-like protein members (TF, LTF, and MELTF) from different taxa was retrieved from GenBank compared with the present transferrin-like protein from the bigfin reef squid. Alignments were performed using MUSCLE in SeaView^45^. The phylogenetic tree was constructed on the SeaView browser by maximum likelihood using PhyML with the LG model of substitution combined with the neighbor-joining interchange method^45^. The accession numbers of the sequences used for analysis are listed in Table S1.

### RNA analysis

First-strand cDNA was used for quantitative real-time PCR analyses (qPCR), which were performed as described previously^44^. *Elongation factor 1 alpha* (*Ef1a*; GenBank accession No. MG924746) was used as an internal control to normalize the gene expression levels. Specific qPCR primers for *Tf* and *Ef1a* are listed as follows (*Ef1a*: 5′-CCAGGTGACAATGTTGGTTTC-3′ and 5′-GTCTCTTTGGGTGGGTTATTCT-3; *Tf*: 5′-GTGGTCCTTGATGGTGGAGATATCT-3′ and 5′-GCCTTTACTACAGCGACAGCATAGT-3). The amplicon sizes of *Ef1a* and *Tf* were 101 bp and 119 bp, respectively. qPCR was performed using the GeneAmp 7500 Sequence Detection System (Applied Biosystems, Foster City, CA, USA) with SYBR Green Master Mix (Applied Biosystems, Vilnius, Lithuania). The thermal cycling conditions were as follows: one cycle of 95 °C for 10 minutes, then 40 cycles of 95 °C for 15 seconds, and 60 °C for 1 minute. The PCR specificity was confirmed by a single melting curve in unknown samples and standards. No signal was detected in non-template controls by qPCR. The data were calibrated according to the relative Ct value with standard and samples, as in our previously study^44^. The relative expression value of target gene in all samples was normalized to *Ef1a*, and the highest value of the target gene was defined as 100%.

### *In situ* hybridization

Digoxigenin-11-UTP (DIG)-labeled antisense and sense probes were synthesized using the cDNA fragments of *Tf* (nucleotides 1374–1996). For *in situ* hybridization (ISH), the methodology described previously was employed^44^. Paraffin-embedded ANGs were sliced (6 μm thickness), deparaffined, rehydrated, and processed for ISH. The sections were incubated with DIG-labeled RNA probes at 60 °C overnight. The mRNA expression was detected with ANG-preadsorbed alkaline phosphatase-conjugated sheep anti-DIG antibody (11093274910, Roche) and colorized by NBT/BCIP Detection System (11681451001, Roche).

### Recombinant TF production

The ORF of *Tf* was inserted into the T&A Expression Vector (Yeastern Biotech, Taipei, Taiwan). To amplify the ORF of *Tf*, the PCR primers 5′-ATGGCTTTGTCGGTGGCT-3′ and 5′-TGCAAGATAATTTATAAAAGTGAATAGCAT-3′ were used. The BL21 (DE3) strain of *E*. *coli* was used as a host for the recombinant construct. The *E*. *coli* were grown at 37 °C and the recombinant TF (rTF, with an external six histidine at the C-terminal region) was induced by the addition of 1 mM of isopropyl-β-D-thiogalactopyranoside (IPTG), followed by culture at 37 °C for 3 hours in LB medium. The insoluble particles of rTF formed in *E*. *coli* were dissolved in 5% SDS with PBS and then used for Western blot (WB) analysis.

### Antiserum production

A guinea pig polyclonal antibody was generated against the C-terminal peptide fragment (C-HLSQIKNPNEFLGKD, amino acid 637–651) of the bigfin reef squid TF. The guinea pigs were immunized with bovine serum albumin (BSA)-conjugated polypeptide. The antisera were prepared by Yao-Hong Biotechnology, Inc. (New Taipei, Taiwan). The specificity of purified anti-TF antibody was confirmed by WB analysis and used for immunohistochemical staining and immunofluorescence staining.

### Western blot analysis

ANG protein was extracted with PBS (containing 25 μM phenylmethylsulfonyl fluoride, pH 7.4) or T-PER™ Tissue Protein Extraction Reagent (Pierce Biotechnology, Rockford, IL, USA) (RIPA solution containing cOmplete™ Mini Protease Inhibitor Cocktail; Roche, Penzberg, Germany). For WB analysis, we followed the methodology described previously^44^. To detect the rTF and TF, anti-His tag antibody (R930-25, Invitrogen; diluted 1:1000 with 1.5% nonfat milk powder) and anti-TF antibody (diluted 1:5000 with 1.5% nonfat milk powder) were used. For secondary antibody reaction, alkaline phosphate-conjugated goat anti-mouse IgG antibody (31320, Thermo Fisher Scientific, Waltham, MA, USA; diluted 1:10000 with 1.5% nonfat milk powder) and alkaline phosphate-conjugated goat anti-guinea pig IgG antibody (YH80013, Yao-Hong Biotechnology; diluted 1:10000 with 1.5% nonfat milk powder) were used for the anti-His tag antibody and anti-TF antibody, respectively. To visualize the immunereactivity, the NBT/BCIP Detection System (B1911, Sigma-Aldrich, St. Louis, MO, USA) was used. To validate the specificity of anti-TF antibody, the antigen-preadsorbed anti-TF antibody (1 mg/ml of the antigenic peptide) was used as a reference.

### Immunohistochemical staining and immunofluorescence staining

Immunohistochemical (IHC) and immunofluorescence (IF) staining were performed according to previously described methods^44^. The paraffin embedded ANGs were sectioned (6 μm thickness), deparaffined, rehydrated, and processed for IHC and IF. The sections were treated with HistoVT One (Nacalai Tesque) to expose the antigens of the target protein. To detect TF in bigfin reef squid, anti-TF antibody (diluted 1:1000 with 1.5% nonfat milk powder) was used. For secondary antibody reactions, biotinylated goat anti-guinea pig antibody (BA-7000, Vector Laboratories Inc., Burlingame, CA; diluted 1:1000 with 1.5% nonfat milk powder) and Alexa Fluor 488-conjugated goat anti-guinea pig secondary antibody (A11073, Thermo Fisher Scientific; diluted 1:1000 with 1.5% nonfat milk powder) were used for IHC and IF staining, respectively. For IHC staining, immunoreactivity was amplified with an ABC kit (avidin-biotin, Vector) and visualized by 3,3′-diaminobenzidine (DAB, Sigma-Aldrich). For IF staining, DAPI was used to label the nucleus. To validate the specificity of the anti-TF antibody, antigen-preadsorbed anti-TF antibody (1 mg/ml of the antigenic peptide) was used as a reference.

### Data analysis

The data are shown as mean ± standard deviation (SD). Shapiro-Wilk test was used to test the normal distribution (variance > 0.5 indicating normality). Games-Howell test was used to check the homogeneity of different groups. Student’s *t*-test was used to check the significance of difference between two groups. Statistical Package for the Social Sciences (SPSS) software with a one-way ANOVA, followed by a Games-Howell test (homogeneity of variance < 0.05), was used to check the significance of difference among three or more groups. In both cases, *P* < 0.05 indicated a significant difference.

## Supplementary information

Supplementary Information
