# Supplementary material for: The Characteristics and Expression Profile of Transferrin in the Accessory Nidamental Gland of the Bigfin Reef Squid during Bacteria Transmission
Source: Sci Rep. 2019 Dec 27;9:20163. doi: 10.1038/s41598-019-56584-8 (PMC6934447; doi:10.1038/s41598-019-56584-8)
Supplement: Supplementary file 1 — Supplementary Information [file 41598_2019_56584_MOESM1_ESM.pdf]

The Characteristics and Expression Profile of Transferrin during Bacteria Transmission in the Accessory Nidamental Gland of the Bigfin Reef Squid

Hau-Wen Li[1], Chih Chen[1], Wei-Lun Kuo[1], Chien-Ju Lin[1], Ching-Fong Chang[1,2] and Guan-Chung Wu[1,2]*

[1]Department of Aquaculture, National Taiwan Ocean University, Keelung, Taiwan
[2]Center of Excellence for the Oceans, National Taiwan Ocean University, Keelung, Taiwan

***Correspondence:**
Guan-Chung Wu
Department of Aquaculture
National Taiwan Ocean University
gcwu@mail.ntou.edu.tw

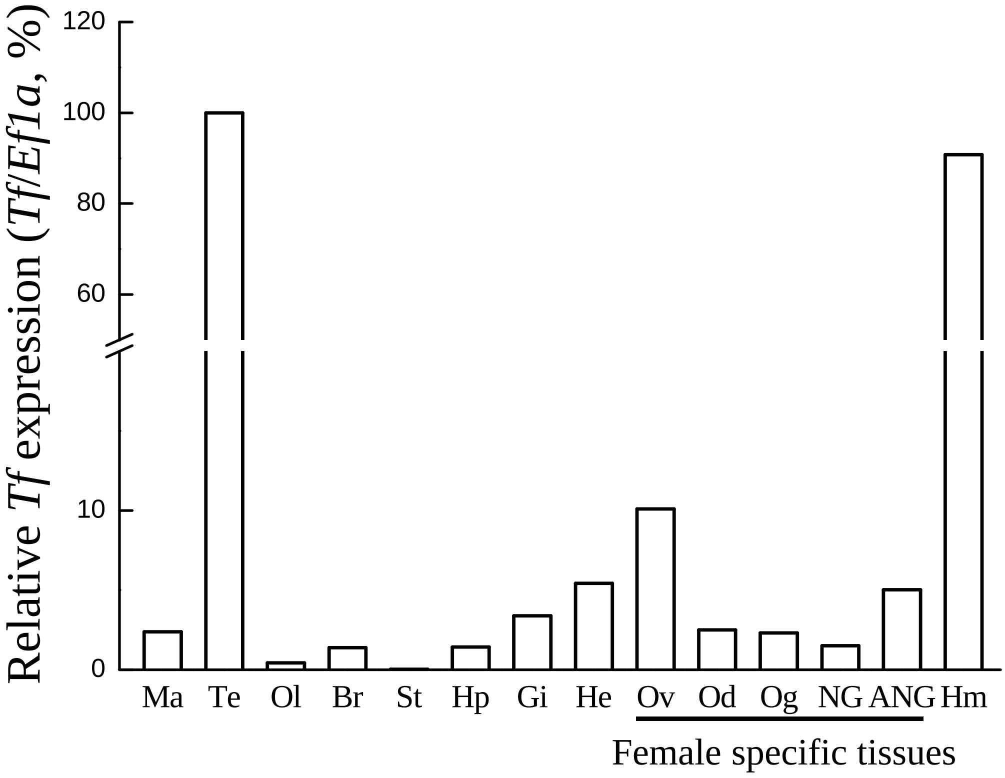

Supplementary Figure 1

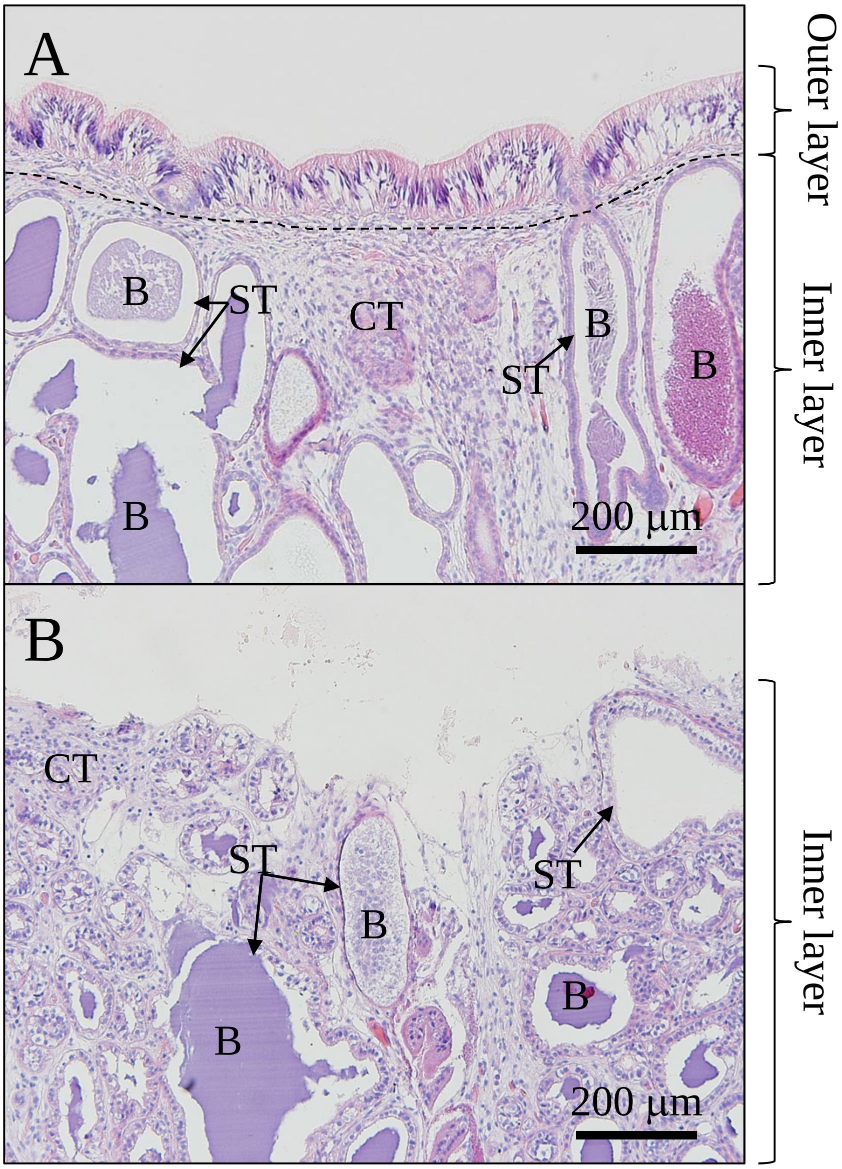

Supplementary Figure 2

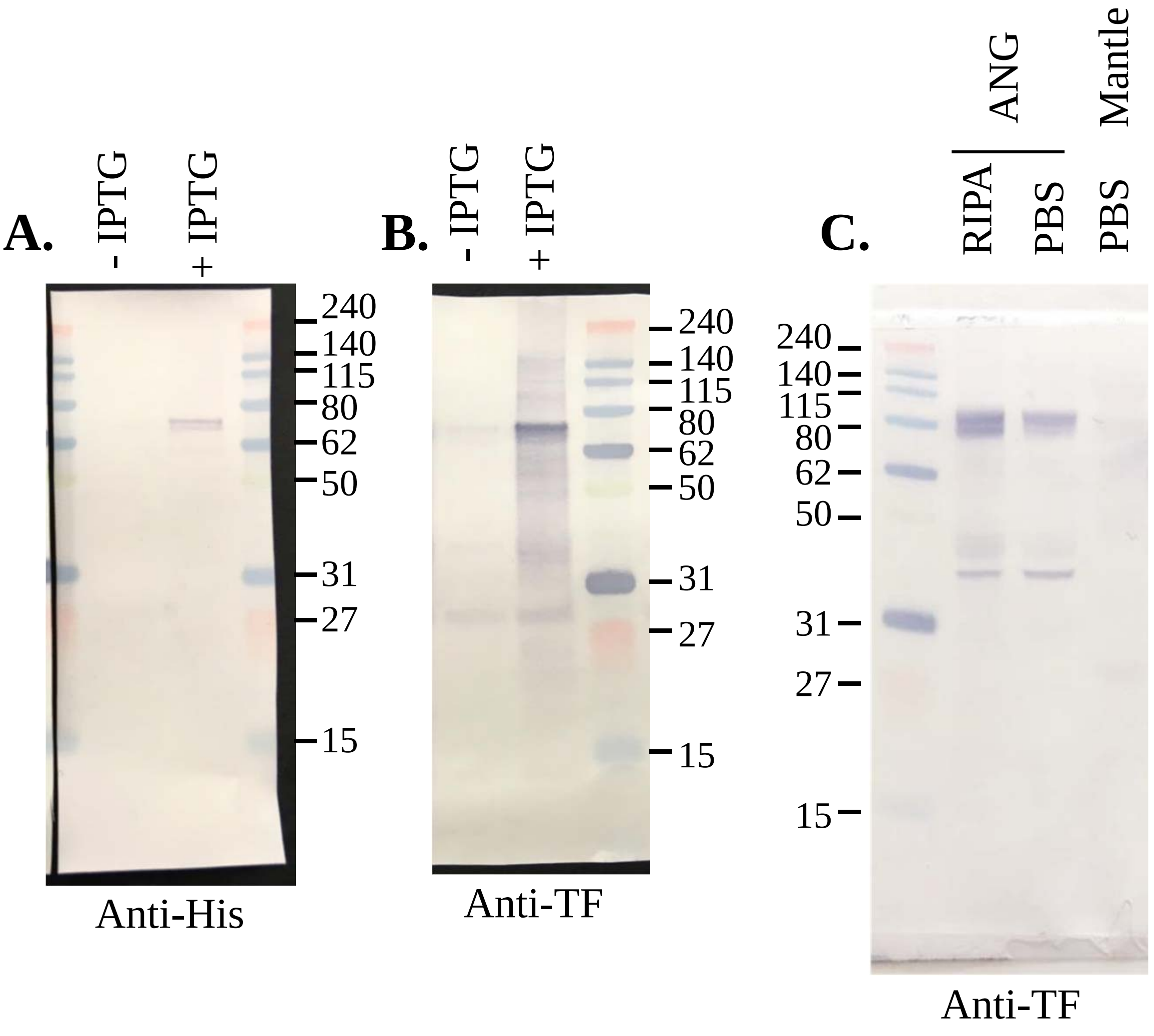

Supplementary Figure 3

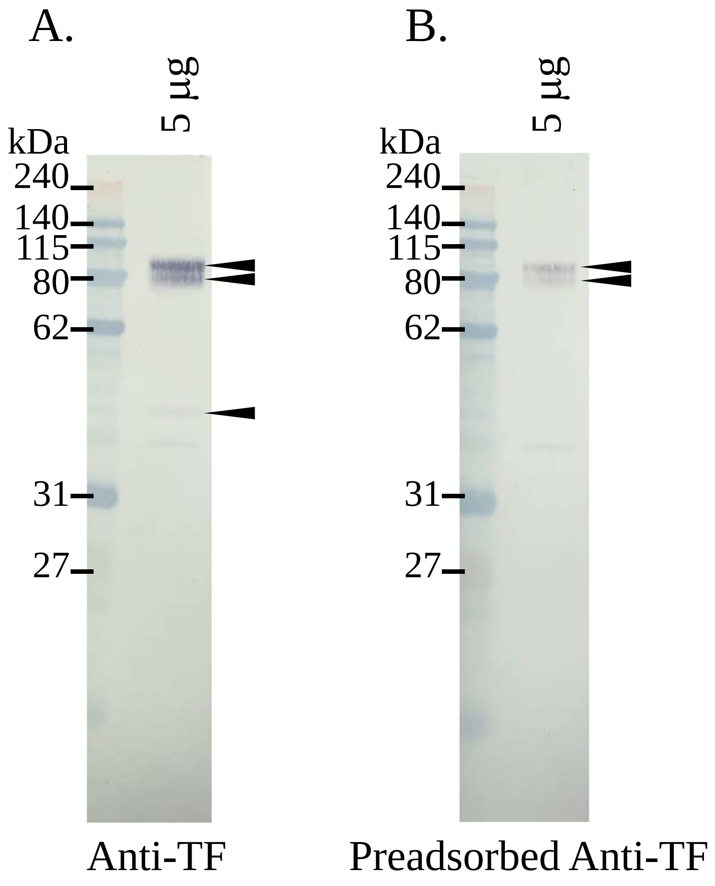

Supplementary Figure 4

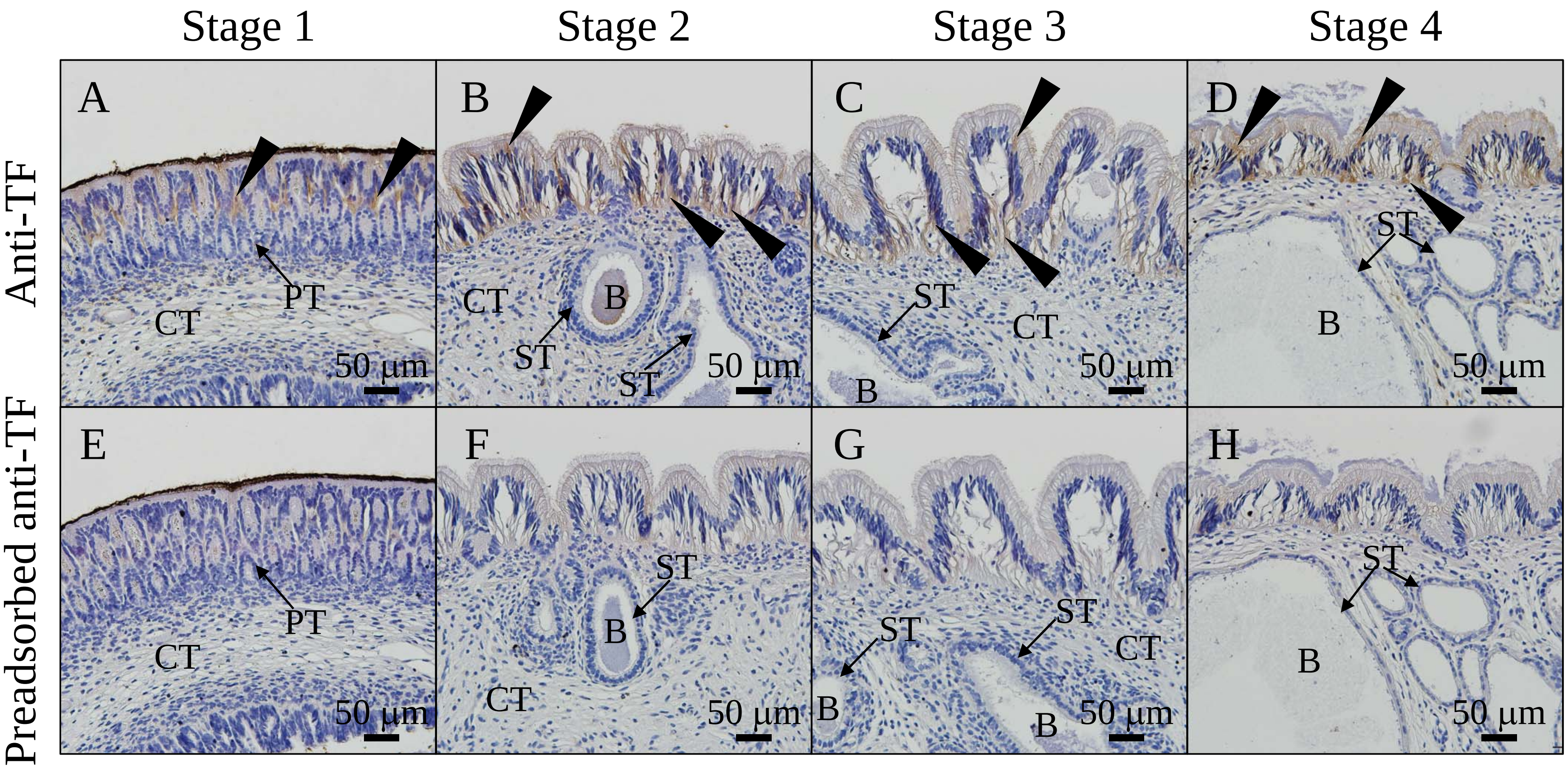

Supplementary Figure 5

Supplemental Figure Legends

Supplemental Figure 1. Expression of *Tf* in various tissues of bigfin reef squid. A mature female squid was used to analyze the gene expression in various tissues, based on duplicate qPCR assay for each tissue.. Differences between the various tissues were normalized with respect of *Ef1a* gene expression, and the highest relative value of *Tf* gene was defined as 100%. Ma, mantle; Te, tentacles; Ol, optic lobe; Br, brain; St, stomach; Hp, hepatopancreas; Gi, gills; He, heart; Ov, ovary; Od, oviduct; Og, oviducal gland; NG, nidamental gland; ANG, accessory nidamental gland; Hm, hemocytes.

Supplemental Figure 2. The full sample length Western blot (WB) in Figure 5. (A) rTF not detected by anti-histidine tag antibody (Anti-His) in reference (- IPTG) but detected in IPTG-induced samples (+ IPTG). (B) rTF not detected by anti-TF antibody (Anti-TF) in reference (- IPTG) and IPTG-induced samples (+ IPTG). (C) WB detected the signals of TF by anti-TF antibody (Anti-TF) in ANGs. ANG protein was extracted with PBS and RIPA. B, bacteria; CT, connective tissue; ST, secondary tubule.

Supplemental Figure 3. Histology of whole versus outer layer-excised ANG. (A) The outer and inner layers of stage 4 ANG. (B) ANG with outer layer-isolated fully excised ANG.

Supplemental Figure 4. Reference trial for specificity of Western blot (WB) assay for the anti-TF antibody. (A) WB of TF protein using anti-TF antibody. (B) WB of TF protein using antigen-preadsorbed anti-TF antibody. The black arrowheads indicate the TF signals.

Supplemental Figure 5. Immunohistochemical (IHC) staining of TF protein at different developmental stages of ANG. (A-D) IHC staining of TF protein using anti-TF antibody in different ANG stages (stages 1-4). (E-H) IHC staining of TF protein using antigen-preadsorbed anti-TF antibody in different ANG stages (stages 1-4). The black arrowheads indicate the TF. B, bacteria; CT, connective tissue; In, intestine; IS, Ink sac; PT, primary tubule; ST, secondary tubule.
